# Multi-omics integration and Mendelian randomization reveal the mechanisms and experimental validation of curcumin targeting the RXRA–PI3K/AKT axis to enhance cisplatin sensitivity in gastric cancer

**DOI:** 10.3389/fonc.2026.1791971

**Published:** 2026-04-15

**Authors:** Xiaoran Sun, Na Wu, Xue Bai, Xiang Zhang, Rui Wang, Shuai Du, Li Liu, Duo Li

**Affiliations:** 1 Department of Gastroenterology, The First Affiliated Hospital of Hebei North University, Zhangjiakou, China; 2 Endoscopy Center, The First Affiliated Hospital of Hebei North University, Zhangjiakou, China

**Keywords:** cisplatin resistance, curcumin, gastric cancer, Mendelian randomization, PI3K/AKT signaling pathway, RXRA

## Abstract

**Objective:**

This study aimed to integrate multi-omics analyses with genetic causal inference to identify key genes associated with cisplatin resistance in gastric cancer and to evaluate the potential mechanism by which curcumin enhances cisplatin sensitivity through relevant pathways.

**Methods:**

Cisplatin resistance–related transcriptomic datasets(GSE14210 and GSE31811) and a gastric cancer single-cell transcriptomic dataset (GSE183904) were obtained from the Gene Expression Omnibus(GEO)database. Differential expression analysis was performed to identify resistance-associated differentially expressed genes(DEGs),followed by GO and KEGG enrichment analyses. Putative curcumin targets were collected and intersected with DEGs to obtain candidate genes. Mendelian randomization (MR) analysis was conducted using the TwoSampleMR framework to evaluate the genetic association between RXRA expression and gastric cancer risk, with robustness and sensitivity analyses based on multiple MR methods. RXRA expression was further evaluated, along with pathway activity assessment using GSEA and GSVA, and molecular docking was performed to explore the potential binding of curcumin to RXRA. *In vitro* experiments were performed using the cisplatin-resistant gastric cancer cell lineNCI-N87/DDP. Drug effects and chemosensitization under combination treatment were assessed by CCK-8 assays, synergy was evaluated using the combination index(CI),and changes in key proteins in thePI3K/AKT pathway were measured by Western blotting.

**Results:**

A total of 595 DEGs associated with cisplatin resistance were identified. Functional enrichment analyses indicated that these DEGs were mainly involved in extracellular matrix remodeling and adhesion, secretion and vesicular transport, and signaling pathways including PI3K-Akt.The intersection of curcumin targets with DEGs highlighted RXRA as a key candidate gene. MR results indicated that genetically predicted increased RXRA expression was significantly associated with elevated gastric cancer risk (OR = 4.216,95%CI:1.201–14.797,P=0.025). GSEA and GSVA suggested that high RXRA expression was associated with altered activity of pathways related to lysosome, proteasome, oxidative phosphorylation, and the pentose phosphate pathway. Single-cell analysis indicated that RXRA was mainly expressed in tissue stem cells and fibroblasts. Molecular docking predicted a feasible interaction between curcumin and RXRA. *In vitro* experiments demonstrated that curcumin inhibited the viability of resistant cells and showed a synergistic trend when combined with cisplatin. Western blotting revealed decreased p-PI3K and p-AKT levels following curcumin treatment, supporting an inhibitory effect on the PI3K/AKT pathway.

**Conclusion:**

These findings highlight RXRA as a candidate gene associated with cisplatin resistance–related programs in gastric cancer. Curcumin may enhance cisplatin sensitivity by influencing RXRA-associated transcriptional networks and suppressing PI3K/AKT signaling. This study provides new candidate targets and experimental evidence for mechanistic investigation and combination treatment strategies to overcome cisplatin resistance in gastric cancer.

## 1 Introduction

Gastric cancer is one of the most prevalent and lethal malignancies of the digestive system worldwide (1). For patients with advanced disease, cisplatin-based combination chemotherapy remains an important treatment strategy (2–5); however, acquired resistance is common and often leads to diminished therapeutic response, increased risk of recurrence, and poor prognosis (3, 6). The development of resistance involves multilayered mechanisms, including enhanced DNA damage repair, evasion of apoptosis, metabolic reprogramming, and adaptation to the tumor microenvironment (7–9). Given the complexity of its molecular basis, there is an urgent need to further clarify key driving events and to propose translatable intervention strategies.

The PI3K/AKT signaling pathway is a central hub governing tumor cell survival, proliferation, and stress adaptation, and is closely associated with chemoresistance (10–12). This pathway can also cooperate with networks such as extracellular matrix (ECM) remodeling and focal adhesion/integrin signaling to sustain invasive phenotypes and therapeutic resistance (13–15). Natural products have attracted substantial interest as sources of chemosensitizers and agents capable of reversing drug resistance (16–18). Curcumin, a polyphenolic compound extracted from turmeric (19, 20), exhibits multi-target antitumor activity and has been reported to modulate multiple signaling pathways, including PI3K/AKT, suggesting potential value in reversing chemotherapy resistance (21, 22). Nonetheless, the key upstream regulatory nodes of curcumin in the context of cisplatin-resistant gastric cancer, its cell type–specific relevance, and causal-level evidence remain to be systematically elucidated.

In this study, we integrated transcriptomic data from multiple cohorts with single-cell transcriptomic data to systematically identify key molecules associated with cisplatin resistance in gastric cancer, and further leveraged Mendelian randomization to improve the reliability of candidate-gene prioritization. Centering on the putative curcumin–nuclear receptor transcriptional regulatory hub–PI3K/AKT axis, we performed pathway and functional validation and evaluated the chemosensitizing effect of curcumin combined with cisplatin in a cisplatin-resistant cell model, together with its underlying molecular basis. This work aims to provide more interpretable and translationally relevant evidence to support mechanistic dissection and chemosensitization strategies for cisplatin resistance in gastric cancer.

## 2 Materials and methods

### 2.1 Overall research design

To investigate the molecular basis of cisplatin resistance in gastric cancer and the potential chemosensitizing effect of curcumin, we adopted a cross-layer analytical framework combining transcriptomic analysis, genetic causal inference, structural prediction, and experimental validation. The study was designed to progressively refine candidate mechanisms by integrating complementary evidence across multiple data layers rather than relying on a single joint statistical model.

Bulk transcriptomic datasets related to cisplatin resistance (GSE14210 and GSE31811) were analyzed to identify differentially expressed genes and resistance-associated pathways. To link these transcriptional alterations with potential pharmacological modulation, resistance-associated genes were intersected with curated curcumin targets, yielding a small set of biologically plausible candidates. Mendelian randomization analysis was subsequently applied to evaluate genetic evidence supporting the involvement of prioritized genes in gastric cancer risk, highlighting RXRA as a key candidate.

Single-cell transcriptomic data (GSE183904) provided cellular context for RXRA expression across tumor and stromal populations. Structural feasibility of a curcumin–RXRA interaction was explored through molecular docking, and functional relevance was further examined using cisplatin-resistant NCI-N87/DDP cells, where the effects of curcumin on drug sensitivity and PI3K/AKT signaling were experimentally evaluated.

### 2.2 Data acquisition and preprocessing

Bulk transcriptomic datasets related to gastric cancer chemoresistance (GSE14210 and GSE31811) were downloaded from the Gene Expression Omnibus (GEO) database, and the gastric cancer single-cell transcriptomic dataset GSE183904 was also obtained. The standard workflow in the limma package was applied for preprocessing (e.g., background correction and quantile normalization). For integrated analyses across multiple bulk datasets, probe identifiers were first mapped to HGNC gene symbols, and the datasets were merged after taking the intersection of shared genes across platforms. Batch-effect correction was subsequently performed to mitigate platform-related variation and reduce the risk of batch-driven differential signals (23). The effectiveness of batch correction was evaluated using principal component analysis (PCA), and the distributions of samples before and after correction are shown in **Supplementary Figure 1**.

### 2.3 Differential expression analysis and visualization

Differential expression analysis was performed between the resistant and sensitive groups. The analysis was conducted using limma-based modeling: a group design matrix (resistant vs. sensitive) was constructed, linear models were fitted, and variance was robustly estimated using empirical Bayes moderation. Differentially expressed genes (DEGs) were defined using the threshold of |logFC| > 0.3 and FDR < 0.05. In drug-resistance studies, relevant regulatory alterations are often subtle but consistent across datasets. Therefore, a relatively permissive logFC threshold was applied while controlling for multiple testing using FDR.

### 2.4 GO/KEGG functional enrichment analysis

Functional enrichment analyses were performed for the identified DEGs using the clusterProfiler package (24). Gene Ontology (GO) enrichment was conducted across the three domains: Biological Process, Cellular Component, and Molecular Function, along with Kyoto Encyclopedia of Genes and Genomes (KEGG) pathway enrichment. Multiple-testing correction was performed using the Benjamini–Hochberg method. An adjusted significance threshold of FDR < 0.05 was applied.

### 2.5 Curcumin target collection and candidate gene selection

Potential targets of curcumin were collected from multiple public databases to ensure comprehensive coverage, including TCMSP, SwissTargetPrediction, STITCH, and DrugBank/BindingDB. For TCMSP, targets associated with curcumin were retrieved directly from the database annotation. In SwissTargetPrediction, predicted targets were obtained by submitting the canonical SMILES structure of curcumin, and only targets with a non-zero probability score were retained. For STITCH, chemical–protein interaction data were retrieved using curcumin as the query compound, and interactions with a combined score ≥ 0.4 (medium confidence) were included.

Targets obtained from different resources were merged and standardized at the gene-symbol level using HGNC-approved gene names. Duplicate entries were removed to generate a nonredundant list of putative curcumin targets. The resulting target set was then intersected with the differentially expressed genes (DEGs) identified from chemoresistance-related transcriptomic datasets to obtain candidate genes potentially involved in both curcumin pharmacological activity and gastric cancer cisplatin resistance. These intersecting genes were retained for subsequent prioritization and downstream analyses.

### 2.6 Mendelian randomization analysis

A TwoSampleMR framework was used to evaluate the potential causal relationship between RXRA and gastric cancer risk (25). Mendelian randomization (MR) analysis was performed using the TwoSampleMR framework implemented in R (version 4.2.1). Genetic instruments for RXRA expression were derived from the Genotype-Tissue Expression (GTEx) v8 eQTL dataset. SNPs significantly associated with RXRA expression (p < 5 × 10^-8^) were selected as instrumental variables and clumped for linkage disequilibrium (LD) using the European reference panel (r² < 0.01, window = 10 Mb). Outcome summary statistics were obtained from the FinnGen R11 GWAS release for gastric cancer–related endpoints, including finngen_R11_C3_STOMACH_ADENO_EXALLC, finngen_R11_C3_STOMACH_EXALLC, and finngen_R11_C3_STOMACH_NEUROENDOCRINE_EXALLC. Effect alleles were harmonized between exposure and outcome datasets, and palindromic SNPs with ambiguous strand orientation were removed. The inverse-variance weighted (IVW) method was used as the primary estimator, with MR-Egger and weighted median methods applied as complementary analyses to evaluate robustness. Heterogeneity (Cochran’s Q), horizontal pleiotropy (Egger intercept), funnel plots, and leave-one-out analyses were used to assess potential violations of MR assumptions. Effect estimates were reported as odds ratios (ORs) with 95% confidence intervals (CIs) (26). SNPs significantly associated with RXRA expression (according to predefined eQTL significance criteria) were selected as instrumental variables and clumped for linkage disequilibrium (LD) (r² < 0.01, window = 10 Mb). Effect alleles were aligned and harmonized, and palindromic SNPs with ambiguous strand orientation were removed. Instrument strength was assessed to reduce the risk of weak-instrument bias. The MR framework relies on the assumptions that genetic variants are associated with the exposure, are not linked to confounders, and influence the outcome only through the exposure. The inverse-variance weighted (IVW) method was used as the primary estimator, complemented by MR-Egger and the weighted median method to assess robustness. Heterogeneity (Cochran’s Q), horizontal pleiotropy (Egger intercept), funnel plots, and leave-one-out analyses were used to evaluate pleiotropy and single-SNP influence. Because only a limited number of significant RXRA-associated variants were available under the predefined criteria, five SNPs were retained as instrumental variables. Effect estimates were reported as odds ratios (ORs) with 95% confidence intervals (CIs), and p < 0.05 was considered statistically significant.

### 2.7 RXRA expression analysis, GSEA, and GSVA

RXRA expression levels were compared between resistant and sensitive samples, and distributions were visualized using box plots. For gene set enrichment analysis (GSEA), samples were divided into high- and low-expression groups based on the median RXRA expression level, and enrichment was evaluated using a pre-ranked gene list with KEGG-related gene sets. For gene set variation analysis (GSVA), pathway activity scores at the sample level were quantified using the GSVA package based on KEGG pathways, and differences in pathway scores between high and low groups were compared and visualized using bar plots.

### 2.8 Molecular docking

The 2D structure of curcumin (PubChem CID: 969516) was retrieved from the PubChem database, and its 3D structure was generated using ChemOffice 2019 (PerkinElmer, USA) and saved in mol2 format. The crystal structure of human RXRA was obtained from the RCSB Protein Data Bank (PDB ID: 1FM9, resolution: 2.1 Å), which contains the ligand-binding domain of RXRA. Protein preprocessing was performed using PyMOL 2.5 (Schrödinger, LLC), including removal of water molecules, ions, and co-crystallized ligands. Polar hydrogens were added and Gasteiger charges were assigned using AutoDock Tools 1.5.6, and the processed receptor and ligand structures were saved in PDBQT format. Rotatable bonds of the ligand were defined during ligand preparation. Molecular docking was performed using AutoDock Vina 1.2.3. The docking grid box was centered on the RXRA ligand-binding pocket based on the coordinates of the co-crystallized ligand. The grid center was defined at (x = 10.3, y = 23.6, z = 15.8) with box dimensions of 24 × 24 × 24 Å, ensuring coverage of the entire ligand-binding cavity. Docking was conducted using an exhaustiveness parameter of 8, and the top-ranked binding pose with the lowest predicted binding energy was selected as the representative binding mode. Docking results were visualized and analyzed using Discovery Studio Visualizer 2019 (BIOVIA, Dassault Systèmes) and PyMOL 2.5 to characterize potential interactions, including hydrogen bonds, hydrophobic contacts, and π–cation interactions, and to identify residues involved in the predicted binding interface.

### 2.9 Single-cell transcriptomic analysis

Single-cell transcriptomic analysis was performed using the gastric cancer dataset GSE183904. Data processing and analysis were conducted in R (version 4.2.1) using the Seurat package (v4.3.0) following a standard single-cell analysis workflow. Quality control was first applied to remove low-quality cells. Cells with extremely low or high gene counts and those with a high proportion of mitochondrial transcripts were excluded to reduce potential technical artifacts. After filtering, gene expression values were normalized using the NormalizeData function with the default log-normalization method. Highly variable genes were identified using FindVariableFeatures, and the data were subsequently scaled using ScaleData. Principal component analysis (PCA) was performed based on the highly variable genes to reduce dimensionality. The top principal components were used to construct a shared nearest-neighbor graph using FindNeighbors, followed by graph-based clustering with FindClusters. Two-dimensional visualization of the cell clusters was generated using Uniform Manifold Approximation and Projection (UMAP) implemented through the RunUMAP function.

Cell-type annotation was performed based on canonical marker genes commonly used in single-cell studies together with marker genes provided within the Seurat analysis framework. Major cell populations identified in the dataset included tissue stem cells, fibroblasts, endothelial cells, T cells, monocytes, and natural killer (NK) cells. Marker gene expression patterns were examined across clusters to ensure consistency with known biological signatures. To evaluate the cellular distribution of RXRA, its expression pattern was visualized on the UMAP embedding using FeaturePlot, and expression levels across annotated cell types were compared using VlnPlot. These analyses were used to characterize the cell-type–specific expression landscape of RXRA within the gastric cancer microenvironment.

### 2.10 Cell culture and drug-resistant cell line

The human gastric cancer cell line NCI-N87 was obtained from the Cell Bank of the Chinese Academy of Sciences (Shanghai). Cells were cultured in RPMI-1640 medium supplemented with 10% (v/v) fetal bovine serum, 100 U/mL penicillin, and 100 U/mL streptomycin, and maintained at 37 °C in a humidified incubator with 5% CO_2_. The cisplatin-resistant cell line NCI-N87/DDP was established previously in the laboratory by stepwise dose escalation *in vitro* and maintained under low-dose cisplatin to stabilize the resistant phenotype. Before experiments, cells were recovered briefly in cisplatin-free medium to reduce potential interference from residual drug.

### 2.11 Cell viability and synergy evaluation

Cell viability was evaluated using the Cell Counting Kit-8 (CCK-8; Dojindo Laboratories, Kumamoto, Japan) according to the manufacturer’s instructions. NCI-N87/DDP cells were seeded in 96-well plates at a density of 5 × 10³ cells per well and allowed to attach for 24 h. Curcumin (Sigma-Aldrich, St. Louis, MO, USA) was dissolved in dimethyl sulfoxide (DMSO) to prepare stock solutions and further diluted in culture medium to the desired working concentrations. The final concentration of DMSO in all treatment groups was maintained at ≤0.1%, and corresponding solvent control groups containing the same concentration of DMSO without drug treatment were included. Cells were treated with different concentrations of curcumin and incubated for 48 h. Subsequently, 10 μL of CCK-8 reagent was added to each well and incubated for 1–2 h at 37 °C. Absorbance was measured at 450 nm using a microplate reader (Bio-Rad, Hercules, CA, USA). Cell viability was calculated relative to the solvent control group, and IC_50_ values were estimated using nonlinear regression analysis in GraphPad Prism (version 9.0).

For combination treatment experiments, cells were exposed to different concentration combinations of cisplatin (DDP; Sigma-Aldrich) and curcumin (CUR) for 48 h, and inhibition rates were calculated based on CCK-8 absorbance values. Drug interactions were evaluated using CompuSyn software (version 1.0) based on the Chou–Talalay method, which calculates the combination index (CI). CI values were interpreted as follows: CI < 1 indicates synergistic effects, CI = 1 indicates additive effects, and CI > 1 indicates antagonistic effects. Each experiment was performed with at least three independent biological replicates, and each condition included three technical replicate wells.

### 2.12 Western blot

After 48 h of drug treatment, cells were harvested and lysed in RIPA lysis buffer (Beyotime Biotechnology, Shanghai, China) supplemented with protease and phosphatase inhibitor cocktails (Roche, Basel, Switzerland). Lysates were centrifuged at 13,000 rpm for 20 min at 4 °C, and the supernatants were collected. Protein concentrations were determined using the BCA Protein Assay Kit (Thermo Fisher Scientific, Waltham, MA, USA). Equal amounts of protein (20–30 μg per lane) were separated by SDS-PAGE and transferred onto PVDF membranes (Millipore, Billerica, MA, USA). Membranes were blocked with 5% bovine serum albumin (BSA) in TBST for 1.5 h at room temperature and then incubated overnight at 4 °C with the following primary antibodies: PI3K (Cell Signaling Technology, CST, #4249; 1:1000); Phospho-PI3K (CST, #4228; 1:1000); AKT (CST, #9272; 1:1000); Phospho-AKT (Ser473) (CST, #4060; 1:1000); GAPDH (CST, #5174; 1:5000). After washing with TBST, membranes were incubated with HRP-conjugated secondary antibodies (CST, anti-rabbit IgG, #7074; 1:5000) for 1 h at room temperature. Protein bands were detected using an enhanced chemiluminescence (ECL) detection system (Thermo Fisher Scientific) and visualized using a chemiluminescence imaging system (Bio-Rad). Band intensities were quantified using ImageJ software. Phosphorylated protein levels were normalized to their corresponding total protein levels (p-PI3K/PI3K and p-AKT/AKT), and loading differences were further controlled by normalization to GAPDH.

### 2.13 Statistical analysis

All experiments were independently repeated at least three times, and data are presented as mean ± standard deviation. Comparisons between two groups were performed using an independent-samples t-test. Comparisons among multiple groups were performed using one-way analysis of variance (ANOVA), with *post hoc* multiple comparisons (e.g., Tukey) added as appropriate based on the actual analysis. For non-normally distributed data, Wilcoxon or Kruskal–Wallis tests were applied. All tests were two-sided, and p < 0.05 was considered statistically significant.

## 3 Results

### 3.1 Identification and functional enrichment of DEGs associated with gastric cancer chemoresistance

**Figure 1:**
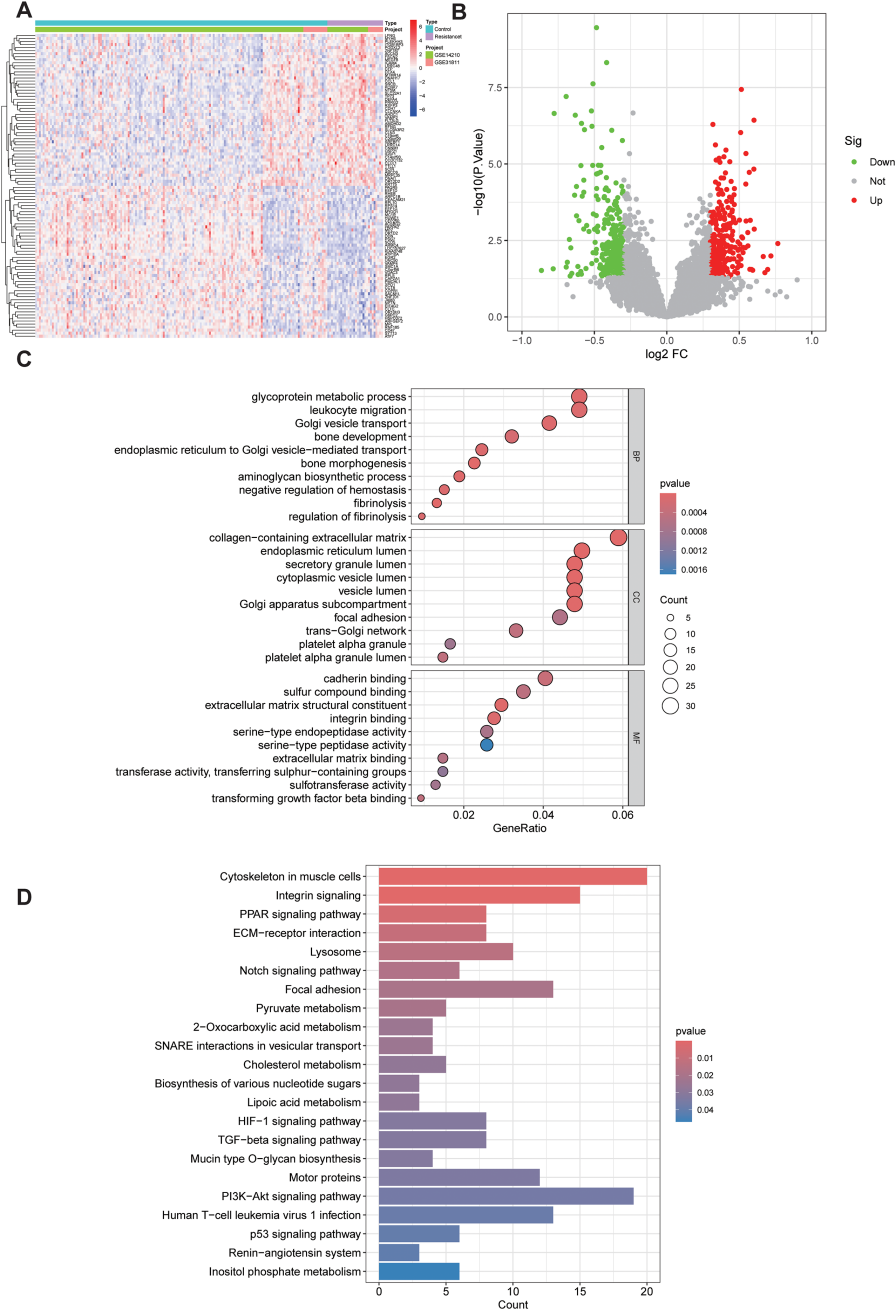
Identification of differentially expressed genes (DEGs) associated with chemoresistance in gastric cancer. **(A)** Heatmap showing the global expression profiles of chemoresistant and chemosensitive samples in the GSE14210 and GSE31811 datasets. **(B)** Volcano plot illustrating the distribution and statistical significance of DEGs. **(C)** Bubble plot of GO enrichment analysis for DEGs. **(D)** Bar plot of KEGG pathway enrichment analysis for DEGs.

Chemoresistant and chemosensitive samples in the GSE14210 and GSE31811 datasets were compared, yielding a total of 595 differentially expressed genes (DEGs), including 282 downregulated and 313 upregulated genes (**Figures 1A, B**). The heatmap showed clear separation between resistant and sensitive groups at the global expression-profile level, and the volcano plot further illustrated the statistical significance and directionality of these DEGs.

GO enrichment analysis indicated that the DEGs were primarily enriched in extracellular matrix remodeling and adhesion-related terms, including collagen-containing extracellular matrix, focal adhesion, and integrin/cadherin binding. Enrichment was also observed in pathways related to vesicle transport and secretion, suggesting that chemoresistance-associated transcriptional alterations may participate in tumor cell–matrix interactions and secretory communication processes.

KEGG analysis showed significant enrichment of DEGs in the PI3K-Akt signaling pathway. Together with pathways such as focal adhesion, ECM–receptor interaction, TGF-β, HIF-1, and Notch, these results delineate a functional network related to cell survival, migration, invasion, and microenvironmental remodeling, supporting the PI3K/AKT axis as a key signaling hub in gastric cancer chemoresistance (**Figure 1D**).

### 3.2 Intersection of curcumin targets and DEGs highlights RXRA with genetic support from MR

**Figure 2:**
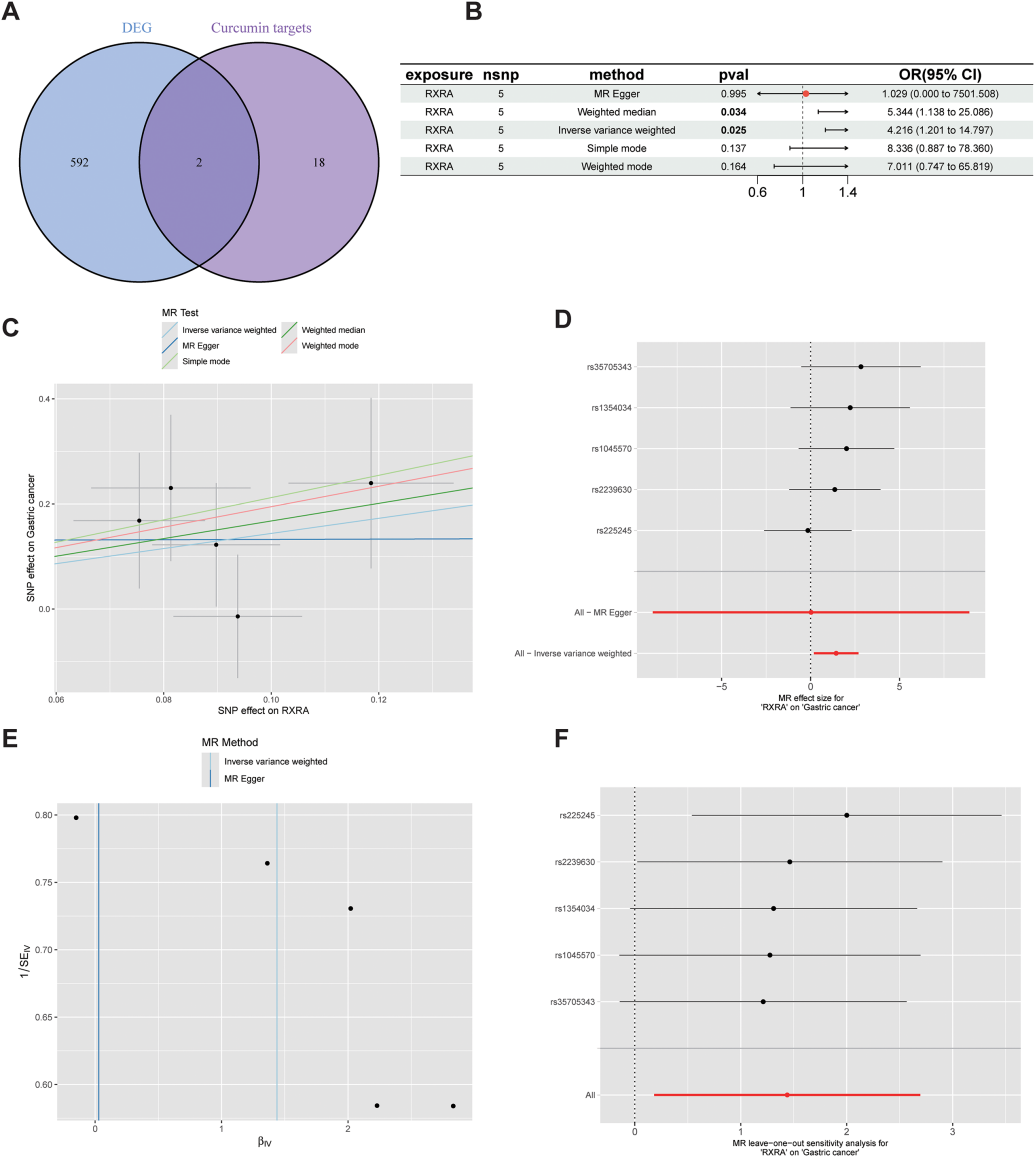
Mendelian randomization (MR) analysis of candidate genes. **(A)** Intersection of curcumin targets and DEGs. **(B)** Forest plot of MR estimates for RXRA. **(C–E)** Scatter plot, forest plot, and funnel plot for RXRA, showing the causal effect on gastric cancer risk, the causal estimates of individual SNPs, and the overall heterogeneity, respectively. **(F)** Leave-one-out analysis assessing the influence of each SNP on the overall causal estimate to identify potential driving variants and evaluate result robustness.

Intersecting the putative targets of curcumin with chemoresistance-related DEGs yielded two candidate genes (RXRA and IL1R1). Based on the direction of differential expression in the resistant group together with supportive genetic evidence from MR analysis, RXRA was selected as the primary focus for mechanistic investigation and experimental validation. To evaluate the genetic association between RXRA expression levels and gastric cancer risk, Mendelian randomization (MR) analysis was conducted (**Figure 2**). Using RXRA-associated genetic variants as instrumental variables, five SNPs were included in the MR analysis. The inverse variance weighted (IVW) method indicated that genetically predicted increases in RXRA expression were significantly associated with an elevated risk of gastric cancer (OR = 4.216, 95% CI: 1.201–14.797, P = 0.025). Consistently, the weighted median method showed an effect in the same direction (OR = 5.344, 95% CI: 1.138–25.086, P = 0.034), further supporting a potential positive association between genetically predicted RXRA expression and gastric cancer risk. In contrast, MR-Egger, simple mode, and weighted mode methods did not reach statistical significance, although their effect directions were generally consistent with the IVW estimate.

### 3.3 Molecular features of RXRA: differential expression, pathway enrichment, and docking evidence

**Figure 3:**
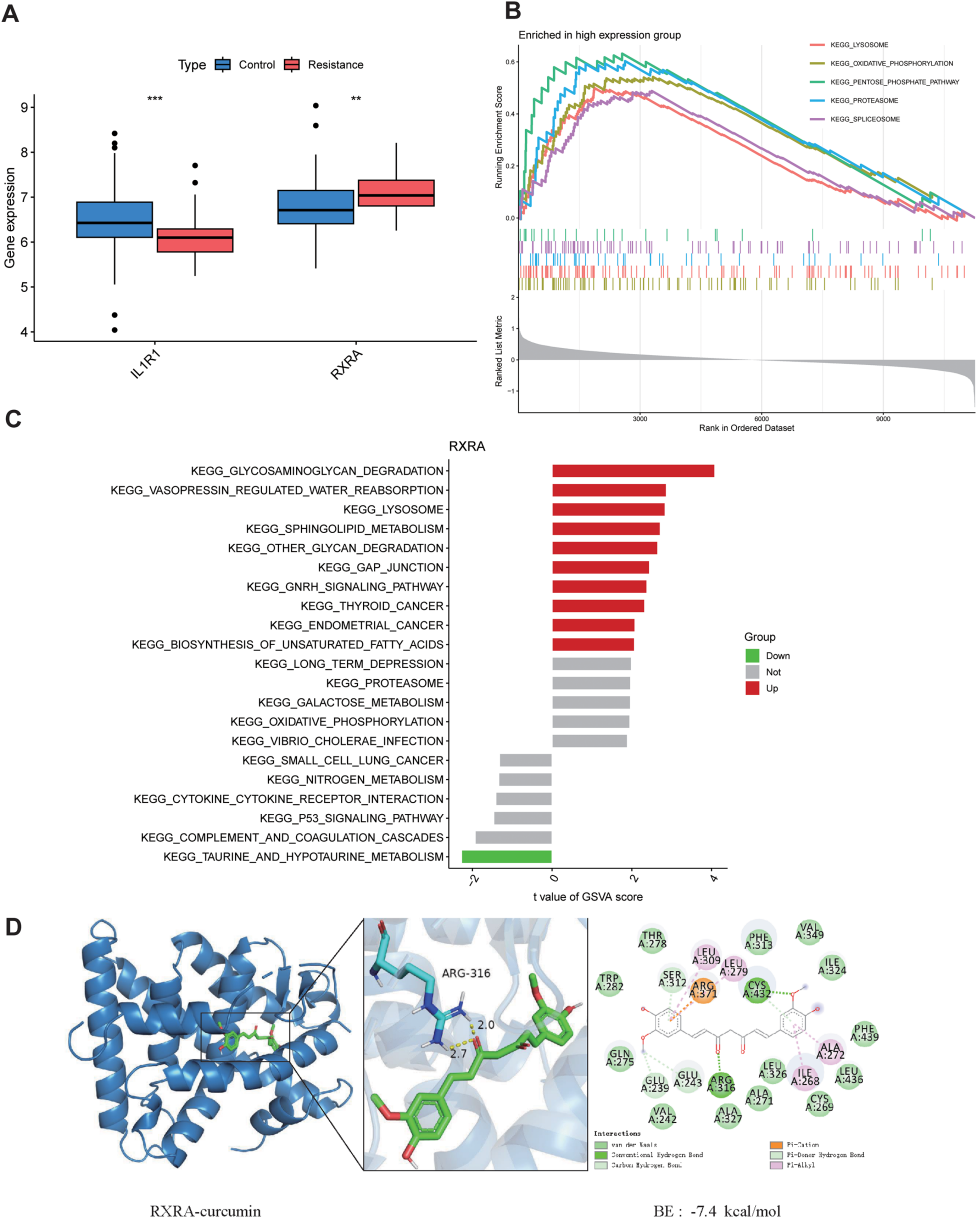
Molecular characteristics of RXRA. **(A)** Box plot of RXRA expression. **(B)** GSEA enrichment analysis in the RXRA high-expression group. **(C)** GSVA results for RXRA-associated pathways. **(D)** Molecular docking between RXRA and curcumin.

RXRA showed significant differential expression between groups (**Figure 3A**). GSEA suggested that the RXRA-associated transcriptional program was closely related to protein homeostasis, stress adaptation, and metabolic reprogramming (**Figure 3B**). GSVA further validated alterations in multiple metabolism- and tumor-related pathway activities at the sample level (**Figure 3C**). Molecular docking predicted a plausible binding pose of curcumin within the RXRA ligand-binding pocket, suggesting potential hydrogen-bond and hydrophobic interactions with residues in the receptor domain. These structural predictions provide hypothesis-generating support for a possible curcumin–RXRA interaction (**Figure 3D**).

### 3.4 Single-cell transcriptomics reveals cell-type-specific expression of RXRA

**Figure 4:**
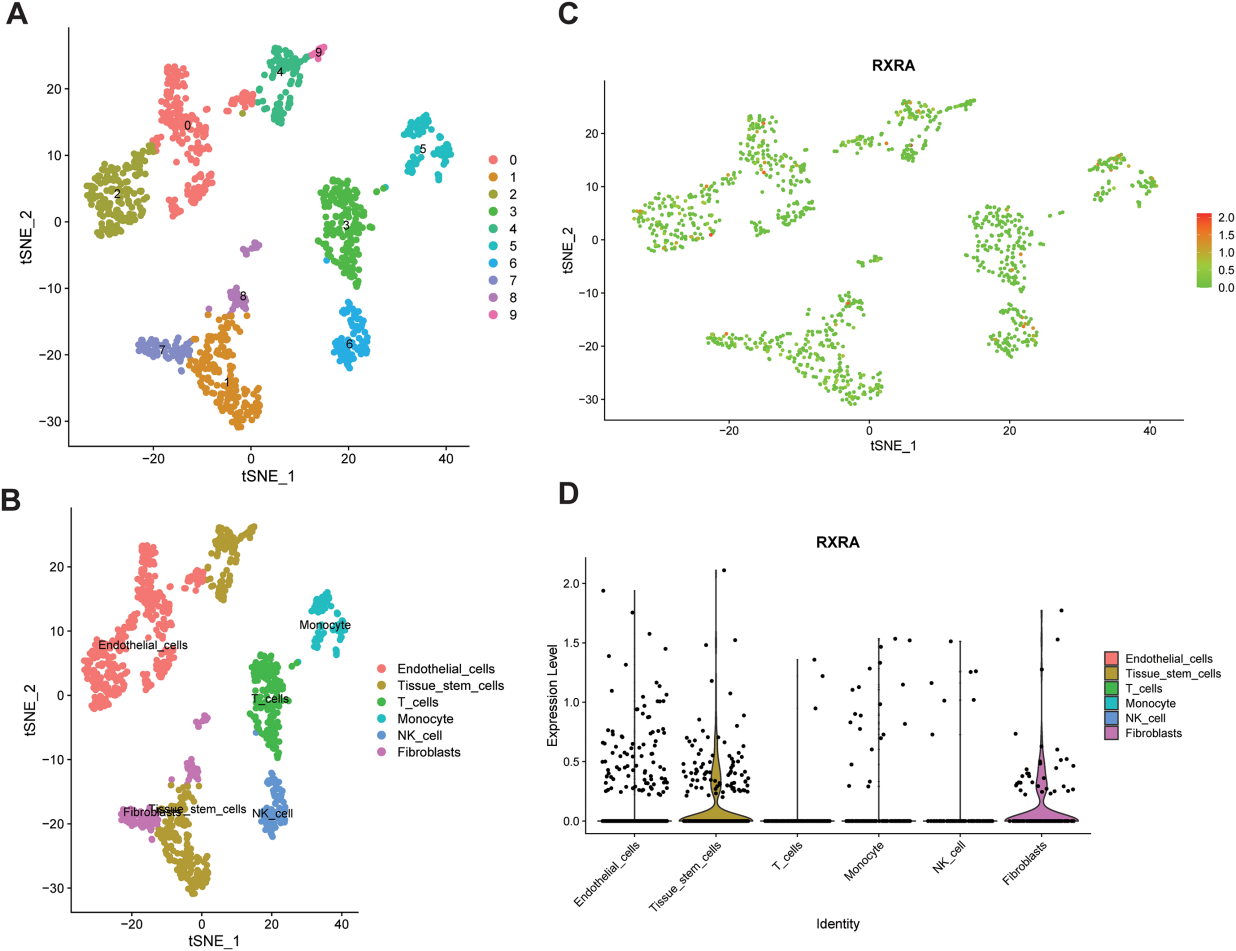
UMAP visualization of single-cell transcriptomic profiles in gastric cancer. **(A)** UMAP projection showing the clustering of major cell populations identified in the GSE183904 dataset. **(B)** Cell-type annotation based on canonical marker genes. **(C)** FeaturePlot showing the distribution of RXRA expression across the UMAP embedding. **(D)** Violin plots comparing RXRA expression levels across annotated cell types.

In the GSE183904 single-cell dataset, UMAP-based clustering and annotation identified major cell populations including tissue stem cells, fibroblasts, endothelial cells, and multiple immune cell types (**Figures 4A, B**). FeaturePlot and violin plots demonstrated relatively higher and more stable RXRA expression in tissue stem cells and fibroblasts, while expression was generally lower in endothelial and immune cell populations (**Figures 4C, D**). These findings suggest that RXRA is preferentially expressed in cell populations associated with tumor stemness and stromal remodeling, providing cellular context for subsequent mechanistic hypotheses, which is consistent with resistance-associated pathways enriched in the differential analyses, such as PI3K-Akt, ECM–receptor interaction, focal adhesion, and TGF-β.

### 3.5 Curcumin and cisplatin exhibit synergistic inhibitory effects in NCI-N87/DDP cells

**Figure 5:**
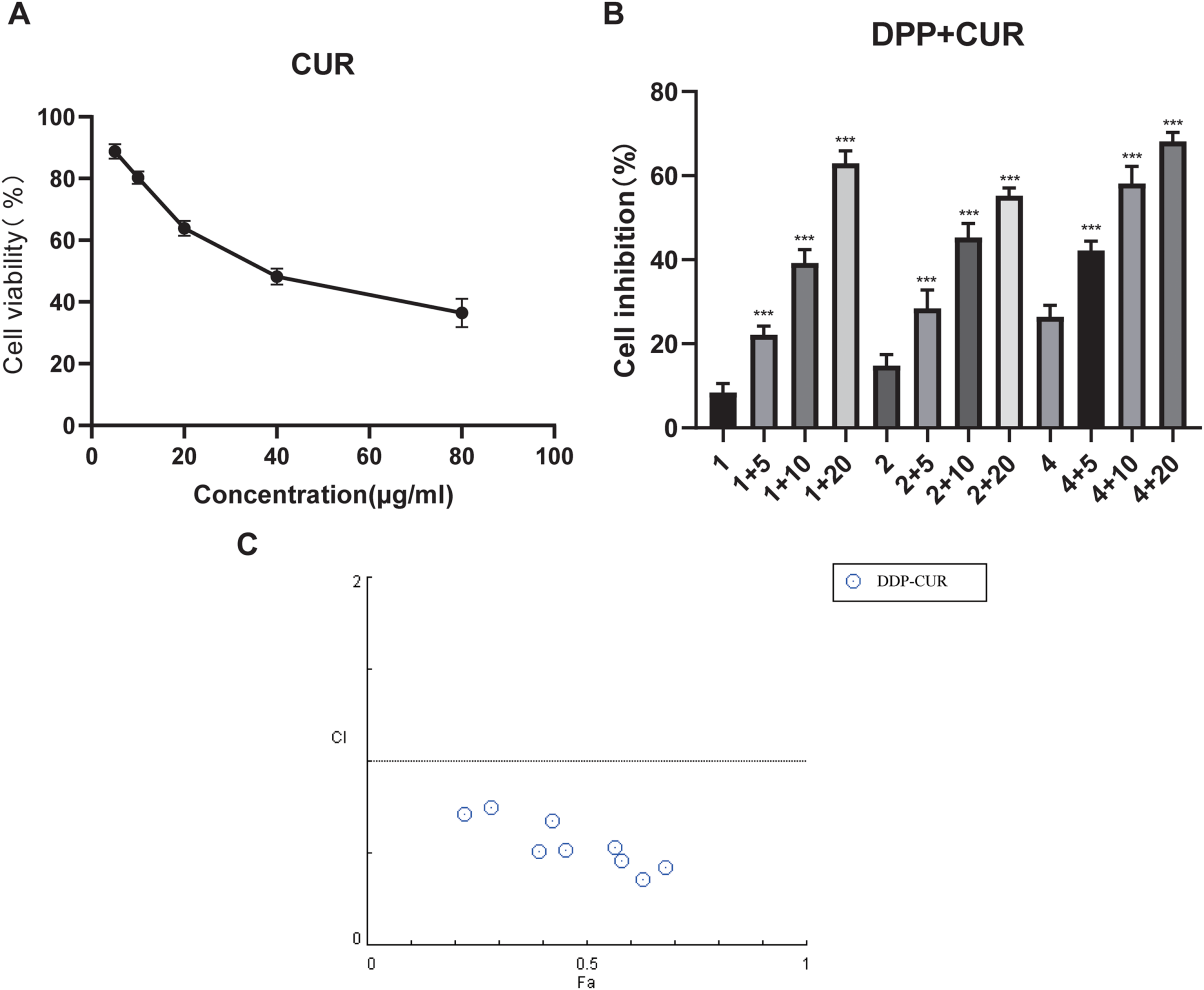
Synergistic effects of curcumin and cisplatin. **(A)** Growth inhibition curve of curcumin in NCI-N87/DDP cells. **(B)** Cell inhibition rates under combined treatment with curcumin and cisplatin. **(C)** Scatter plot of FA–CI values for the combination of curcumin and cisplatin. Data are presented as mean ± SD (n=3). ***P < 0.001 vs. the cisplatin-alone group.

Previous studies have suggested that curcumin can inhibit PI3K/Akt signaling; therefore, its potential to reverse cisplatin resistance in NCI-N87/DDP cells was further evaluated. First, the cytotoxicity of curcumin in NCI-N87/DDP cells was assessed using the CCK-8 assay. The results showed that curcumin decreased cell viability in a dose- and time-dependent manner (**Figure 5A**). The 50% inhibitory concentration (IC50, mean ± SD) of curcumin in NCI-N87/DDP cells was 40.08 ± 4.52 μg/mL.

To assess the interaction between curcumin and cisplatin, the combination index (CI) was calculated. After defining single-agent effects, cisplatin (DDP; 1, 2, 4 μg/mL) and curcumin (CUR; 5, 10, 20 μg/mL) were tested in combination for 48 h. Compared with cisplatin alone, the combination treatment significantly enhanced growth inhibition in NCI-N87/DDP cells (P < 0.0001; **Figure 5B**). CI values were then calculated using CompuSyn software based on the Chou–Talalay equation. The results indicated that most combination points exhibited synergistic effects under the 48 h condition (**Figure 5C**).

### 3.6 Synergy is associated with suppression of the PI3K/AKT pathway

**Figure 6:**
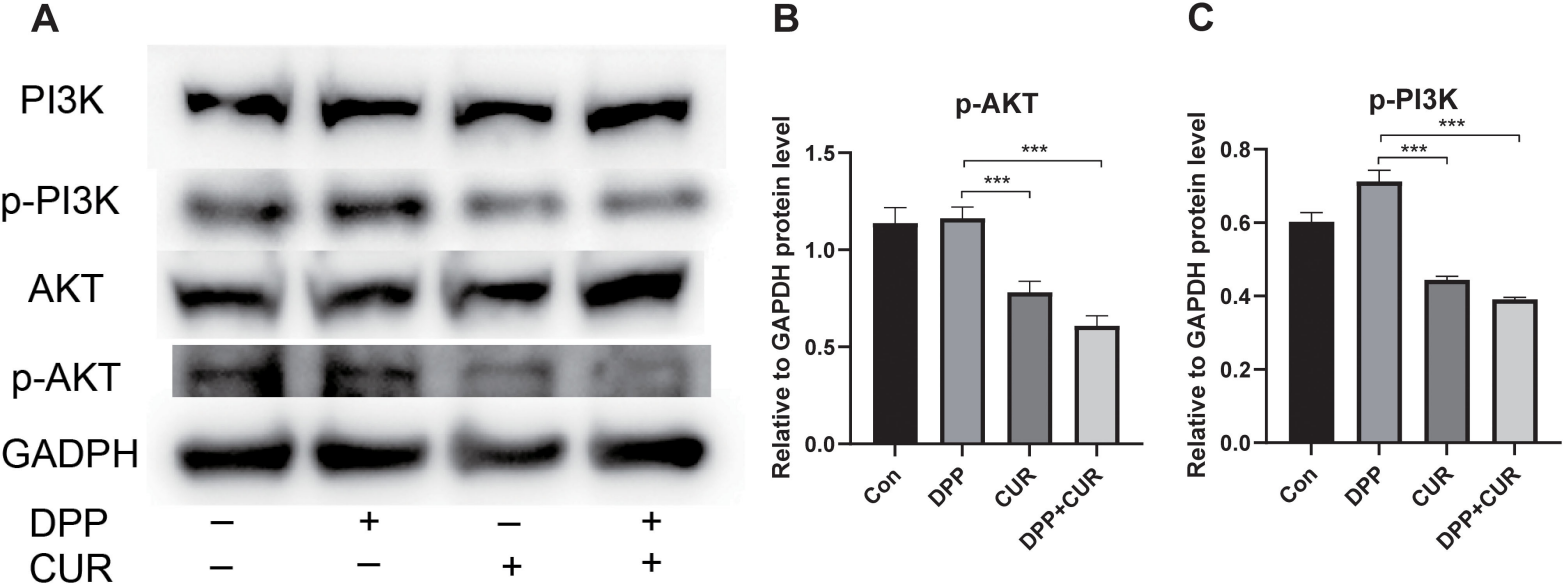
PI3K/AKT protein expression after drug treatment assessed by Western blot. **(A)** Western blot results of PI3K/AKT-related proteins after drug treatment. **(B)** Quantification of p-PI3K protein levels. **(C)** Quantification of p-AKT protein levels. Data are expressed as mean ± SD (n = 3). ***P < 0.001 vs. the control group.

Given the critical role of the PI3K/AKT pathway in cisplatin resistance, Western blotting was performed to examine changes in PI3K/AKT-related proteins in NCI-N87/DDP cells (**Figure 6A**). No significant differences in p-PI3K or p-AKT levels were observed between the control and cisplatin-alone groups (P > 0.05). In contrast, p-PI3K and p-AKT were significantly reduced in the curcumin-alone group and the combination-treatment group (P < 0.001; **Figures 6B, C**). These results suggest that the addition of curcumin suppresses PI3K/AKT pathway activation, which may contribute to the synergistic inhibitory effect observed with cisplatin. Accordingly, it is proposed that CUR enhances cisplatin-induced cytotoxicity in resistant cells by inhibiting activation of the PI3K/AKT signaling pathway.

## 4 Discussion

Gastric cancer is one of the most common malignant tumors of the digestive system worldwide, and platinum-based regimens remain a cornerstone of systemic therapy for patients with advanced disease (27, 28). Cisplatin exerts antitumor effects primarily by inducing DNA damage that triggers cell-cycle arrest and apoptosis; however, acquired resistance is frequent in clinical practice, often resulting in attenuated treatment responses, increased risk of recurrence, and limited survival benefit (29). The development of resistance typically arises from the cooperation of multi-level mechanisms, including altered drug uptake and efflux, enhanced DNA repair, evasion of apoptosis, metabolic reprogramming, and adaptation to the tumor microenvironment (30, 31). Accordingly, there is a pressing need to identify key drivers at a systems level and to propose actionable therapeutic strategies. In this study, we focused on molecular drivers and targetable pathways underlying cisplatin resistance in gastric cancer by integrating differential expression analysis, functional enrichment, genetic causal inference, and *in vitro* pharmacological validation, thereby establishing an evidence chain from candidate-gene prioritization to mechanistic implications and combination-based sensitization.

Enrichment analyses consistently highlighted the PI3K-Akt signaling pathway together with focal adhesion, ECM–receptor interaction, and integrin signaling, supporting a central role for the transmission of adhesion and matrix-derived cues into pro-survival signaling during resistance acquisition (32–34). Focal adhesion and integrin signaling can promote cytoskeletal remodeling and migratory/invasive behavior while enhancing anti-apoptotic capacity and activating PI3K/AKT-associated downstream effects, thereby increasing the likelihood of survival under chemotherapy-induced stress (35, 36). In parallel, GSEA and GSVA pointed to altered activities in lysosome, proteasome, spliceosome, oxidative phosphorylation, and the pentose phosphate pathway, suggesting that resistant cells may adapt to drug pressure by strengthening proteostasis maintenance, post-transcriptional regulation, and energy metabolism/redox balance. Functionally, these processes are tightly coupled to PI3K/AKT-mediated control of metabolism and survival. Therefore, positioning RXRA as a candidate upstream transcriptional regulator provides a testable mechanistic framework that integrates these enriched signals, namely that RXRA may be involved in transcriptional programs linked to PI3K/AKT-associated survival signaling, potentially influencing metabolic and proteostatic adaptation together with adhesion and microenvironmental pathways (37, 38).

By introducing Mendelian randomization, this study provided evidence that is closer to the causal level for candidate-gene support (39). Using RXRA-associated genetic variants as instrumental variables, five SNPs were included in the analysis. The inverse variance weighted approach showed that genetically predicted increases in RXRA expression were significantly associated with elevated gastric cancer risk (OR = 4.216, 95% CI: 1.201–14.797, P = 0.025). Consistently, the weighted median method yielded an effect in the same direction (OR = 5.344, 95% CI: 1.138–25.086, P = 0.034), further supporting a potential positive causal effect of RXRA on gastric cancer risk. In contrast, MR-Egger, simple mode, and weighted mode methods did not reach statistical significance, although their directions were generally consistent with the IVW estimate. Taken together, these findings suggest that increased RXRA expression may be associated with elevated gastric cancer risk at the genetic level, providing supportive genetic evidence for a potential role of RXRA in tumor biology. It should be emphasized that MR reflects long-term effects of genetically predicted exposure; its primary value lies in supporting directionality and causal plausibility rather than directly equating to the short-term magnitude of pharmacologic intervention, and thus it should be interpreted together with mechanistic experiments to form a translationally meaningful closed loop.

Single-cell analyses further supported the above framework by clarifying the cellular sources of RXRA expression. RXRA was mainly expressed in tissue stem cells and fibroblasts, while overall expression was lower in endothelial and immune cell populations. This distribution aligns with the biology of resistance. Tissue stem-like cells are often linked to stemness maintenance, stress tolerance, enhanced drug efflux, and increased repair capacity, forming an important cellular basis for acquired resistance; enrichment of RXRA in this population suggests a potential association with transcriptional reprogramming and metabolic adaptation within resistant subpopulations (40–42). Meanwhile, fibroblasts have been reported to influence tumor-cell adhesion and anti-apoptotic signaling through ECM remodeling and paracrine factors (43–45), and together with PI3K/AKT, TGF-β, and HIF-1 pathways, they may contribute to a microenvironment that is permissive for resistance-related signaling. Accordingly, the cell-type distribution of RXRA expression provides cellular context that may help interpret the enrichment of ECM, focal adhesion, and integrin-related pathways, and it also suggests that future studies may further explore the potential relevance of stem-like niches and tumor–stroma interactions.

Molecular docking analysis predicted a structurally feasible interaction between curcumin and residues within the RXRA ligand-binding domain, suggesting the possibility that curcumin may interact with RXRA and potentially influence RXRA-associated transcriptional programs. *In vitro* experiments further showed that curcumin and cisplatin exerted synergistic inhibitory effects in resistant cells, accompanied by downregulation of p-PI3K and p-AKT, indicating that suppression of PI3K/AKT activation may represent an important component of the sensitization mechanism. Integrating these findings with multi-omics and single-cell evidence supports a coherent working hypothesis: curcumin may influence RXRA-associated transcriptional programs and related adaptive processes involved in adhesion/matrix signaling, metabolism, and proteostasis, thereby contributing to reduced PI3K/AKT-mediated survival advantages and enhanced cisplatin sensitivity. This framework may also help explain why resistant phenotypes often involve parallel, multi-pathway systemic changes and why combination strategies can outperform monotherapy.

Several limitations should be acknowledged. Heterogeneity arising from public cohorts and platform differences is difficult to eliminate completely; even with normalization and batch correction, such variation may affect the stability and reproducibility of candidate-gene identification. MR analyses also depend on instrumental strength, assumptions regarding horizontal pleiotropy, and the tissue relevance of eQTL sources. The genetic instruments used in this study were derived from publicly available eQTL datasets that are not specific to gastric tissue, and regulatory effects may vary across tissues. Although multiple estimators and sensitivity analyses were applied to evaluate robustness, the limited number of available RXRA-associated variants may reduce statistical power and restrict the sensitivity of pleiotropy detection. Moreover, Mendelian randomization provides evidence supporting causal plausibility at the genetic level rather than definitive mechanistic causation. Replication using eQTL resources that more closely match gastric or tumor tissue would therefore further strengthen the inference. The single-cell RNA-seq analysis also has inherent limitations, including a relatively modest dataset size and reliance on canonical marker genes for cell-type annotation, which may introduce uncertainty in defining specific cellular identities. Consequently, the observed enrichment of RXRA expression in fibroblast and stem-like populations should be interpreted as providing cellular context rather than direct evidence of functional dominance. Importantly, although curcumin treatment suppressed PI3K/AKT signaling and enhanced cisplatin sensitivity in resistant cells, the present study did not include direct perturbation experiments targeting RXRA, such as knockdown, overexpression, or rescue assays. Therefore, the proposed RXRA–PI3K/AKT regulatory framework should be interpreted as a working hypothesis supported by multi-layer prioritization rather than definitive mechanistic proof. Finally, while *in vitro* assays can reveal cell-intrinsic mechanisms, they cannot fully recapitulate the complexity of the tumor microenvironment and pharmacokinetic processes; additional validation of the functional necessity of RXRA in organoids, patient-derived xenografts, or animal models will be important for further consolidation.

## 5 Conclusion

This study indicates that transcriptional alterations associated with cisplatin resistance in gastric cancer are primarily enriched in ECM/adhesion remodeling, secretory and vesicular transport processes, and pro-survival networks such as PI3K/AKT signaling. RXRA was identified as a key target, and single-cell analyses showed that it is mainly enriched in tissue stem-like cells and fibroblast populations, supporting its potential involvement in stemness maintenance and matrix-related resistance programs. Together with pathway-level changes in metabolism and proteostasis, these findings suggest that RXRA may couple resistance-associated stress adaptation with survival advantages. Experimental results further indicate that curcumin combined with cisplatin has sensitizing potential and is accompanied by inhibition of PI3K/AKT pathway activation, highlighting the curcumin–RXRA–PI3K/AKT axis as a promising direction for mechanistic studies and translational development.

## Data Availability

The datasets analyzed in this study are publicly available in the GEO repository under accession numbers GSE14210, GSE31811, and GSE183904. Processed data and analysis scripts are available from the corresponding author upon reasonable request.

## Supplementary material

Supplementary Figure 1 Diagnostic evaluation of batch-effect correction for cross-platform transcriptomic integration. Principal component analysis (PCA) plots showing the distribution of samples from the GSE14210 and GSE31811 datasets before and after batch-effect correction. **(A)** PCA visualization before batch correction, where samples are primarily separated according to microarray platform (Affymetrix vs. Agilent), indicating strong platform-driven variation. **(B)** PCA visualization after batch-effect correction, demonstrating a substantial reduction in platform-associated clustering and improved integration of samples across datasets. These results indicate that the applied normalization and batch-correction procedures effectively mitigated platform-related bias while preserving biological variation associated with cisplatin resistance.
