# Supplementary figures and images for: Multi-omics integration and Mendelian randomization reveal the mechanisms and experimental validation of curcumin targeting the RXRA–PI3K/AKT axis to enhance cisplatin sensitivity in gastric cancer
Source: Front Oncol. 2026 Apr 15;16:1791971. doi: 10.3389/fonc.2026.1791971 (PMC13124633; doi:10.3389/fonc.2026.1791971)

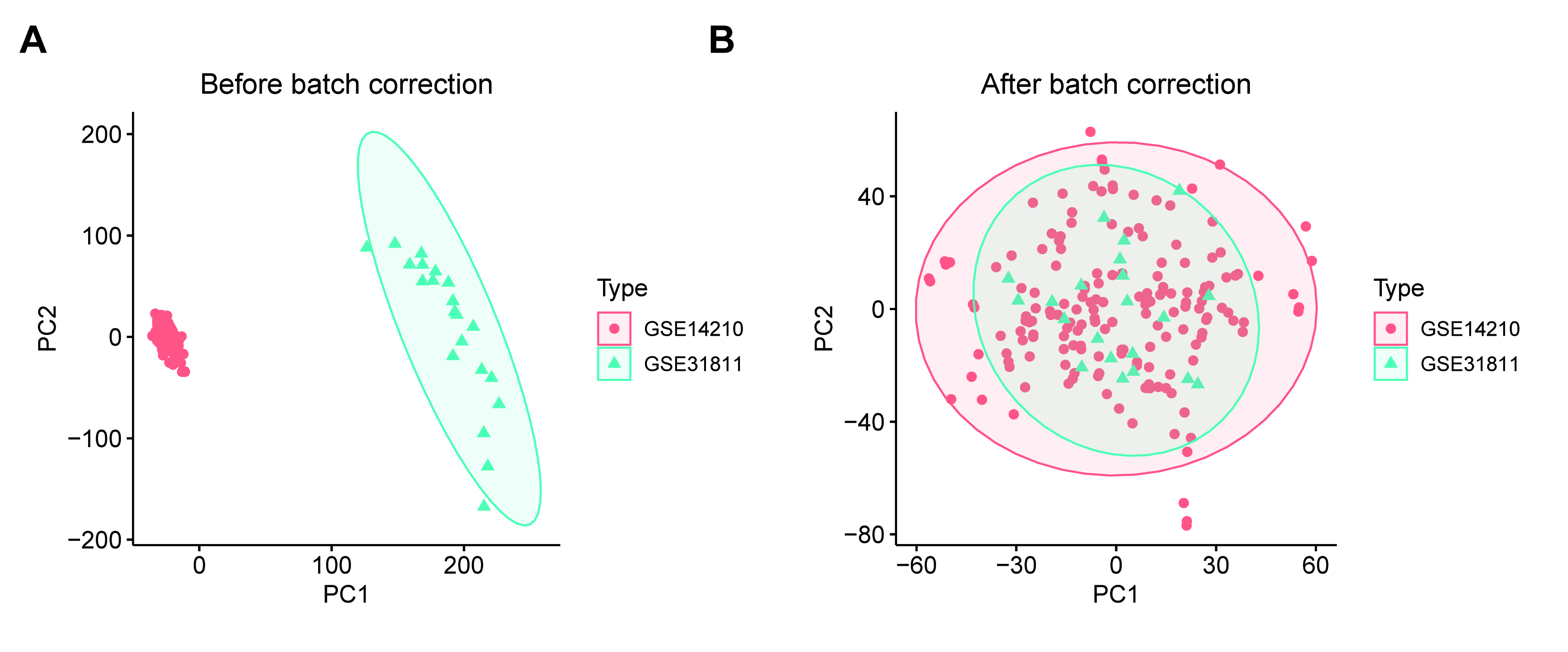

Supplement: Supplementary Figure 1 — Diagnostic evaluation of batch-effect correction for cross-platform transcriptomic integration. Principal component analysis (PCA) plots showing the distribution of samples from the GSE14210 and GSE31811 datasets before and after batch-effect correction. (A) PCA visualization before batch correction, where samples are primarily separated according to microarray platform (Affymetrix vs. Agilent), indicating strong platform-driven variation. (B) PCA visualization after batch-effect correction, demonstrating a substantial reduction in platform-associated clustering and improved integration of samples across datasets. These results indicate that the applied normalization and batch-correction procedures effectively mitigated platform-related bias while preserving biological variation associated with cisplatin resistance. [file Image1.tif]
